# CT scan-derived pectoralis muscle parameters are closely associated with COVID-19 outcomes: A systematic review and meta-analysis

**DOI:** 10.1371/journal.pone.0316893

**Published:** 2025-01-28

**Authors:** Zhang Wen, Tao Wang, Sha Luo, Yiwen Liu

**Affiliations:** 1 Department of Critical Care Medicine, West China Hospital, Sichuan University, Chengdu, China; 2 Department of Pediatric Intensive Care Unit, Chengdu Women’s and Children’s Central Hospital, School of Medicine, University of Electronic Science and Technology of China, Chengdu, China; Fondazione Policlinico Universitario Gemelli IRCCS, ITALY

## Abstract

**Background:**

The relationships between pectoralis muscle parameters and outcomes in patients with coronavirus disease 2019 (COVID-19) remain uncertain.

**Methods:**

We systematically searched PubMed, Embase, Web of Science and the Cochrane Library from 1 January 2019 to 1 May 2024 to identify non-overlapping studies evaluating pectoralis muscle-associated index on chest CT scan with clinical outcome in COVID-19 patients. Random-effects and fixed-effects meta-analyses were performed, and heterogeneity between studies was quantified using the I^2^ statistic. The risk of study bias was assessed using the Newcastle-Ottawa scale. Funnel plots for detecting small-study effects.

**Results:**

A total of 9 studies with 4109 COVID-19 patients were included. The meta-analysis findings revealed a correlation between pectoralis muscle parameters and COVID-19 prognosis. Specifically, patients with higher pectoralis muscle density (PMD) exhibited a lower mortality risk, with an odds ratio (OR) of 0.95 (95% CI: 0.92–0.99). The rate of intubation was lower in COVID-19 patients with a high pectoralis muscle index (PMI) (OR =  0.96, 95% CI: 0.92–1.00).

**Conclusion:**

In summary, a low PMD is associated with a marginally elevated risk of mortality, whereas a decreased PMI represents a risk factor for intubation in COVID-19 patients. These findings suggest that pectoralis muscle parameters on chest CT may be a useful prognostic tool for COVID-19 patients.

## Introduction

The ongoing community-wide spread of COVID-19 and its ensuing pandemic ushered in a myriad of unprecedented clinical dilemmas. COVID-19 symptoms range from mild respiratory infections to severe manifestations such as pneumonia, ARDS, hypoxia, multiorgan failure, and fatality. Various COVID-19 vaccines have demonstrated efficacy in preventing SARS-CoV-2 infections and mitigating COVID-19 severity [1–3]. Although various COVID-19 vaccines have played pivotal roles in reducing the COVID-19 pandemic to an endemic state [4], the emergence of SARS-CoV-2 variants has complicated the situation, potentially significantly diminishing the efficacy of existing vaccines [5]. In addition to vaccines, various therapeutic options, including monoclonal antibodies and antiviral agents, have emerged, yet the strategy for risk stratification among symptomatic individuals to prioritize these treatments remains a matter of ongoing discourse [6,7].

Studies have demonstrated that comorbidities, particularly diabetes, hypertension, and chronic obstructive pulmonary disease, substantially influence the postoperative prognosis of patients with COVID-19, with these patients having a poorer prognosis than those without comorbidities [8,9]. A reduction in muscle mass is closely associated with the progression of multiple diseases [10,11]. Septic patients with muscle mass depletion and dysfunction frequently experience adverse outcomes during their intensive care unit (ICU) stay [12,13]. Recent research has revealed the incidence of acute sarcopenia among COVID-19 survivors [14], yet the long-term ramifications of both COVID-19 and acute sarcopenia remain unknown. In addition, critically ill SARS-CoV-2-infected patients necessitating mechanical ventilation exhibit reduced muscle mass, strength, and mobility during intensive care unit (ICU) confinement [15].

Previous studies have demonstrated that parameters for assessing chest skeletal muscle mass can be derived from the analysis of patients’ chest CT scans [16]. The chest skeletal muscle area can be calculated from CT images at the level of the twelfth thoracic vertebra. Studies have reported no significant correlation between the chest skeletal muscle area and mortality in patients with COVID-19 [17]. The relationships between indicators derived from chest CT-assessed pectoral muscle parameters and prognosis in COVID-19 patients remain unclear and warrant further exploration and investigation. This review aims to assess the correlation between pectoral muscle parameters derived from chest CT and the prognosis of patients with COVID-19.

## Materials and methods

We followed the Preferred Reporting Items for Systematic Reviews and Meta-Analyses (PRISMA) statements [18].

### Data sources and search

We searched PubMed, Embase, Web of Science and the Cochrane Library for eligible studies with no language restrictions. The following search terms were used: “covid-19” or “sars-cov-2” or “severe acute respiratory syndrome coronavirus 2” or “NCOV” or “2019 NCOV” and “pectoralis” or “muscle, pectoralis” or “pectoralis muscle” or “pectoral muscle” or “pectoral muscles” or “pectoralis major” or “pectoralis minor”. The search was updated on 1 May 2024.

### Selection criteria

The focus of this meta-analysis was on the prognostic value of CT-derived pectoralis muscle parameters in COVID-19 patients. Studies were considered eligible for inclusion if they were observational cohort studies (prospective, retrospective cohort or case‒cohort); reported hazard ratios or odds ratios with 95% confidence intervals; and enrolled patients who tested positive for COVID-19 via PCR and underwent either chest or thoracic CT scans without the use of intravenous contrast medium.

Studies were excluded if relevant data on COVID-19 disease outcomes were not available. Publications without original data, such as reviews, editorials and commentaries, were also excluded. Abstracts and conference proceedings were excluded because of incomplete data.

### Data extraction

Two authors independently extracted data for each of the eligible studies. The extracted data included the first author, year of publication, country, setting, study design, country, sample size, personal and clinical characteristics of the participants, and other outcome variables of interest. Where relevant, attempts were made to contact the authors for missing data.

In accordance with the methods of previous studies [19], a line is drawn along the contours of both the pectoralis major and pectoralis minor muscles on chest CT images, delineating the bilateral areas to define the PMA. The PMD was assessed bilaterally on all contrast-enhanced scans, with the mean value then calculated. The PMI was calculated by dividing the PMA by the patient’s height. The skeletal muscle gauge (SMG) is determined by multiplying the PMI by the average muscle density.

### Quality assessment

For the included studies, the Newcastle‒Ottawa Scale (NOS) was used to assess the quality of the studies included in the meta-analysis. This scale evaluates bias risk across three domains: study group selection, group comparability, and exposure/outcome ascertainment. Studies scoring below 4 indicate high risk of bias, those scoring 4–6 suggest intermediate risk, and those scoring 7 or above imply low risk of bias. An article was considered to be of high quality if its quality score was greater than or equal to 6 out of a total score of 9. Funnel plots were used to assess publication bias.

### Statistical analysis

Statistical analyses were conducted via RevMan 5.3 software (Copenhagen: Nordic Cochrane Centre, Cochrane Collaboration, 2011). For every study, ORs and 95% CIs were used to assess the relationships between pectoralis muscle parameters and the prognosis of patients with COVID-19. We pooled ORs for pectoral parameters employing fixed-effects models, transitioning to random-effects models in instances where heterogeneity was observed. Between-study heterogeneity was evaluated via the Q statistic, and inconsistency was quantified via the I^2^ statistic (0–25% indicates no significant heterogeneity, 26–50% indicates low heterogeneity, 51–75% indicates moderate heterogeneity, and >75% indicates high heterogeneity).

## Results

### Study selection

**Fig 1:**
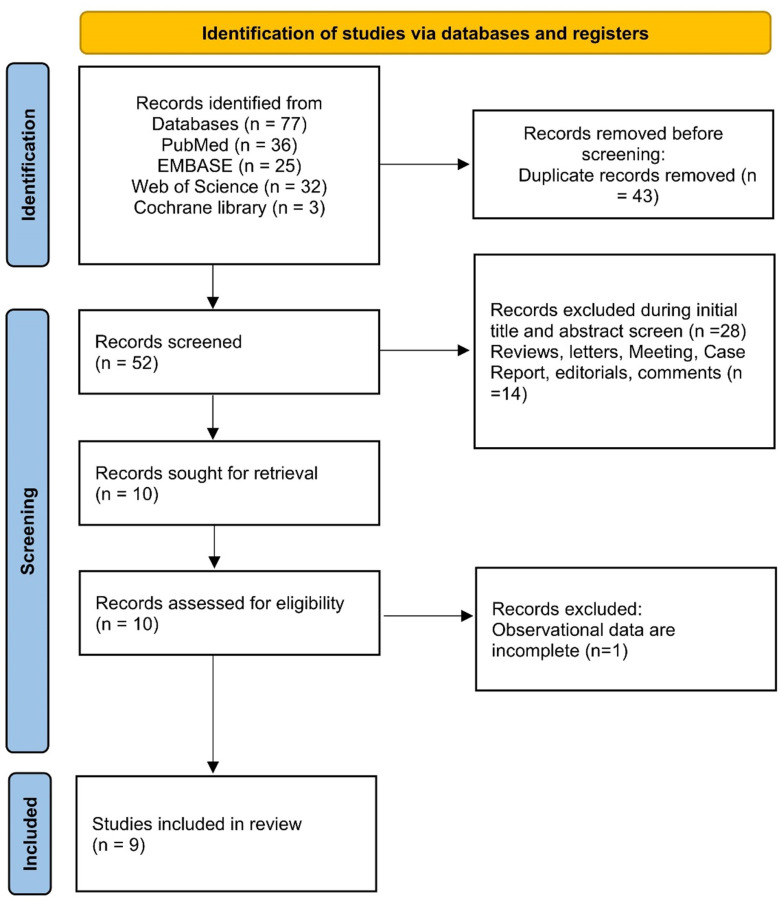
Flow diagram of literature searching and study selection.

Fifty-two records were identified after removing duplicates in our initial systematic search. After title and abstract screening, the full texts of 10 articles were reviewed. One study was excluded because of incomplete data. Finally, 9 retrospective studies that included 3997 COVID-19 patients were included in the systematic review [19–27]. The flow diagram is shown in Fig 1.

### Study characteristics

Among the included studies, 3 were identified from Turkey across three time periods: Period 1 (March 2020April 2020) [25], period 2 (December 2020March 2021) [26], and Period 3 (October 2021 to November 2021) [24]. All COVID-19 cases were confirmed by reverse-transcription polymerase chain reaction testing or highly suspected clinically with a COVID-19 Reporting and Data System (CO-RADS) score ≥4, ruling out other diagnoses [28].

For all patients with an initial admission diagnosis of COVID-19, a chest CT scan was administered within the first 1 to 2 days of their hospital stay as part of the diagnostic workup. In eight studies [19–21,23–27], a chest CT scan was performed within 1–2 days of hospital admission for all patients with an initial diagnosis of COVID-19. In a multicenter study by Nakagawara et al., the associations between changes in muscle parameters and decreased CT density at 3 months and patient prognosis were analyzed in 167 patients [22]. The baseline characteristics of the patients in the included studies are presented in Table 1.

**Table 1:** The descriptive characteristics of the study population.

Authors	Year	Country	Design	No. patients	Age (years)	Sex (Male)	PMA (cm^2^)	PMI (cm^2^/m^2^)	PMD (HU)	Body mass index (BMI), kg/m 2	Diabetes	NOS scores
Ufuk F et al	2020	Turkey	RT	130	48 (18–86)*	50.53%	33.7 (17.2–63)*	12.4 (5.3–21.6)*	–	26.9 (17.1–36.5)*	–	8
Besutti G et al	2021	Italy	RT	318	65.7 (52.8–75.7)*	62.26%	17 (12–21)*	–	34 (27–41)*	–	43 (13.52%)	8
Kardas H et al	2022	Germany	RT	46	64.5 (41–92)*	58.70%	22.8 (9.0–64.0)*	7.7 (3.1–20.9)*	27.0 (4–53.5)*	27.3 (20.6-58.5)*	–	6
Liu Y et al	2023	China	RT	393	–	–	–	–	–	–	–	8
Surov A et al	2023	Italy	RT	1138	54.5 ± 18.8^#^	51.93%	28.91 ± 13.6^#^	10.85 ± 4.47^#^	35.5 ± 13.77^#^	–	–	7
Tekin ZN et al	2023	Turkey	RT	140	62.90 ± 16.6^#^	–	–	–	–	–	–	7
Ufuk F et al	2023	Turkey	RT	238	48 (36–63)*	50.84%	–	–	–	24.6 (23.6-27.5) *	52 (21.85%)	7
van Bakel SIJ et al	2023	Netherlands	RT	578	–	–	–	–	–	–	–	7
Nakagawara K et al	2024	Japan	RT	1128	55.8 ± 16.7^#^	69.32%	–	–	–	24.9 ± 4.9^#^	214(19.00%)	7

Abbreviations: RS, retrospective study; PMA, pectoralis muscle area; PMI, pectoralis muscle index; PMD, Pectoralis muscle density; BMI, Body mass index; NOS, Newcastle-Ottawa Scale; -, data not given.

* Data are shown as IQR.

^#^ Data are shown as mean±SD.

### Primary outcome: mortality

#### Pectoralis muscle area.

The PMA was defined as the total bilateral area encompassing the pectoralis major and minor muscles, as described previously [29]. Five studies [19,23,25–27] that reported on the association between the PMA and mortality risk of COVID-19 patients were included in the synthesis, totaling 2104 individuals.

**Fig 2:**
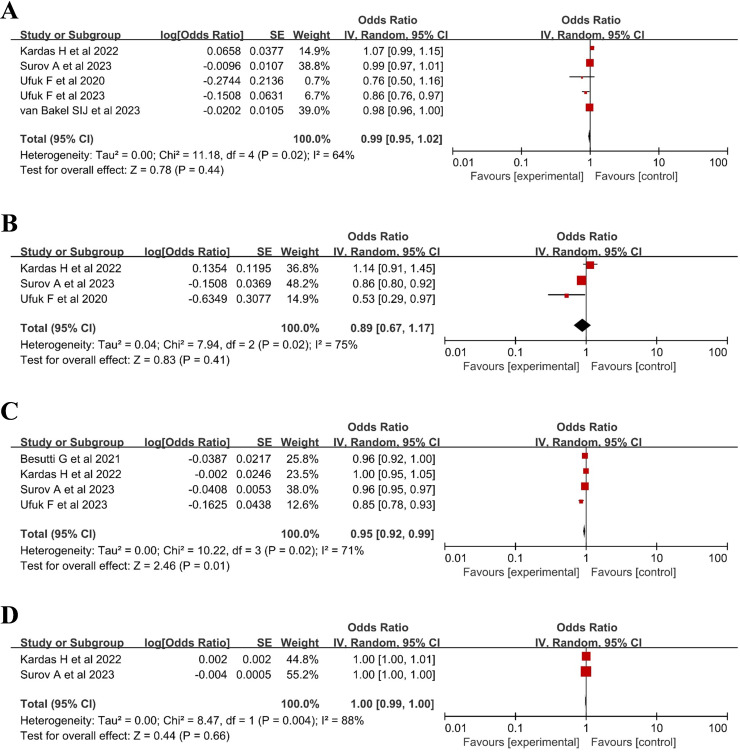
Relationship between mortality and pectoralis muscle parameters in patients with COVID-19. (A) pectoralis muscle area; (B) pectoralis muscle index; (C) pectoralis muscle density; (D) pectoralis muscle gauge.

The meta-analysis results revealed no relationship between PMA and mortality (OR 0.99, 95% CI 0.95–1.02), with high heterogeneity (I^2^  =  64%; P  =  0.02) (Fig 2A). Furthermore, Tekin ZN et al [24]. found that a PMA ≤  2800 was an independent risk factor for death in COVID-19 patients.

#### Pectoralis muscle index.

PMI is calculated by dividing the cross-sectional area of the pectoral muscle by the square of the individual’s height. This metric provides a standardized way to assess the relative size and development of the chest muscles, taking into account the individual’s overall height [30].

A meta-analysis of 3 studies [19,23,25] revealed that the PMI had no significant effect on the mortality risk of COVID-19 patients (OR 0.89, 95% CI 0.67–1.17), with high heterogeneity (I2  =  75%; P  =  0.02) (Fig 2B).

Liu Y et al [21]. evaluated the relationship between high and low PMIs and COVID-19 mortality risk and reported that a low PMI was an independent risk factor for death in COVID-19 patients. As the provided OR value could not be combined with the data from the other included studies, it is described separately here.

#### Pectoral muscle density.

Teigen LM et al. reported that the quantity of the pectoralis muscle is associated with mortality after implantation of a left ventricular assist device [31]. Therefore, four studies reporting the relationship between pectoral muscle density (PMD) and mortality were included in this subgroup analysis.

The meta-analysis results indicated that PMD was associated with mortality (OR 0.95, 95% CI 0.92–0.99), with high heterogeneity (I^2^  =  71%; P  =  0.01). A summary of the findings is shown in Fig 2C. Nakagawara K et al [22]. found that regardless of sex, a lower CT density of the pectoralis muscle was associated with a greater risk of death.

#### Skeletal muscle gauge.

Furthermore, the PMI was multiplied by the average muscle density, as described in the two included studies [19,23], to derive the SMG. The pooled outcome results revealed that decreasing SMG was independent of mortality (OR 1.00, 95% CI 0.99–1.00), with high heterogeneity (I2  =  91%; P  =  0.00007) (Fig 2D).

### Secondary outcome: Intubation

#### PMA.

**Fig 3:**
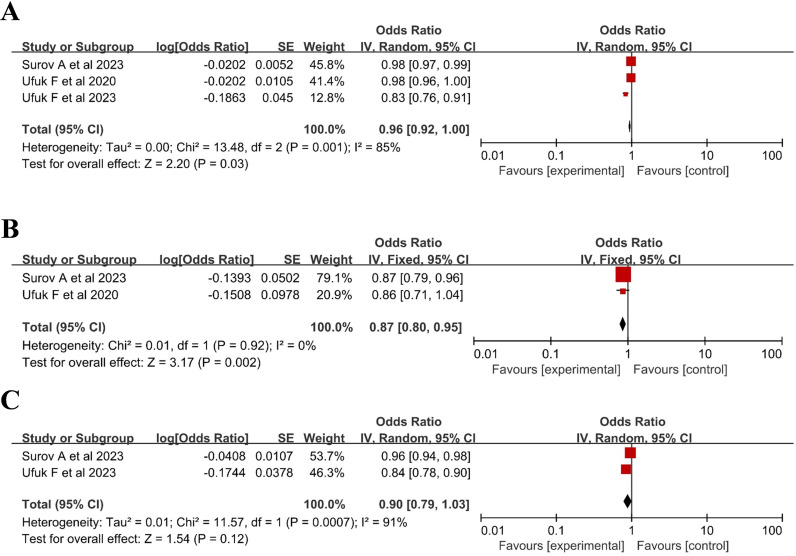
Relationship between intubation rate and pectoralis muscle parameters in patients with COVID-19. (A) pectoralis muscle area; (B) pectoralis muscle index; (C) pectoralis muscle density.

A meta-analysis of 3 studies [23,25,26] investigated the association between the PMA and the need for intubation in COVID-19 patients. The present study revealed no correlation between PMA and intubation (OR 0.96, 95% CI 0.92–1.00), with high heterogeneity observed (I^2^  =  85%; P  =  0.03) (Fig 3A).

#### PMI.

Two articles [23,25] investigated the relationship between the PMI and the need for mechanical ventilation in COVID-19 patients. The meta-analysis results indicated that the PMI was associated with the need for intubation (OR 0.87, 95% CI 0.80–0.95), with low heterogeneity (I^2^ =  0%; P =  0.002) (Fig 3B).

#### PMD.

Combined data from the two studies [23,26] indicated that the PMD was independent of the need for intubation in COVID-19 patients (OR 0.90, 95% CI 0.79–1.03), with high heterogeneity (I^2^ =  91%; P =  0.00007) (Fig 3C).

#### Publication bias.

**Fig 4:**
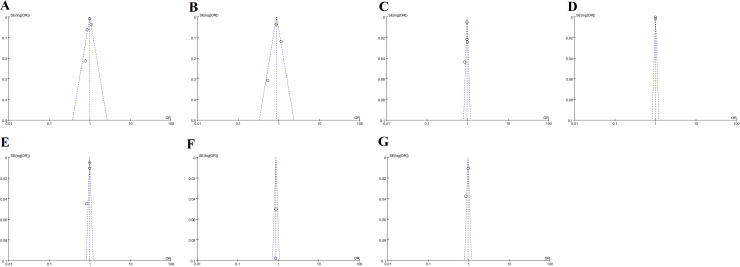
Funnel plots: pectoralis muscle parameters (A pectoralis muscle area, B pectoralis muscle index, C pectoralis muscle density, D pectoralis muscle gauge) on mortality, and (E pectoralis muscle area, F pectoralis muscle index, G pectoralis muscle density) on intubation.

The publication bias in this meta-analysis was assessed via funnel plots, and a summary of the funnel plots is presented in Fig 4. Visual inspection of the funnel plots revealed no substantive evidence of publication bias.

## Discussion

COVID-19, a deadly disease that led to the death of almost 1.1 million people in 2022 [32], can cause rapid respiratory failure, with no specific drug currently available for its treatment [33]. The aim of this study was to assess the associations between pectoralis muscle parameters and the prognosis of COVID-19 patients. Our research findings confirm that lower levels of PMA, PMI, and PMD are associated with an increased risk of patient mortality. Furthermore, the pooled results indicate that lower PMIs and PMDs are correlated with higher intubation rates.

ARDS induced by COVID-19 results in the need for ventilatory support and multisystem damage in critically ill patients, characterized by heightened inflammation, hypermetabolism, and hypercatabolism, primarily affecting the musculoskeletal system [34]. Research by Silva et al. revealed a significant reduction in quadriceps muscle thickness among critically ill patients with COVID-19 during their intensive care unit (ICU) stay [15]. Muscle atrophy in critically ill patients is associated with prolonged hospital stays, extended duration of mechanical ventilation, deep sedation, more severe illness at admission, delirium, and extended periods of bed rest [35]. The degree of decline in muscle mass and function can be used to assess the presence of sarcopenia. Sarcopenia has emerged as a predictive factor for both hospital duration and mortality among critically ill and trauma patients, as evidenced in studies [36,37]. However, the relationship between sarcopenia and the prognosis of patients with COVID-19 remains controversial. Kim et al. quantified the skeletal muscle index at T12 and reported that sarcopenia in COVID-19 patients prolonged hospitalization but did not significantly impact mortality [16]. Feng et al.‘s study of 116 patients revealed no discernible correlation between the paraspinal muscle index at the T12 level and clinical outcomes [38].

Patients diagnosed with COVID-19 are categorized as having mild to severe disease [39]. Thorax CT is a rapid assessment method that can aid in the evaluation of critically ill COVID-19 patients and is utilized to assess the severity and prognosis of COVID-19 [40]. Branea et al. identified alterations in diaphragm thickness through chest CT scan results in critically ill patients diagnosed with COVID-19 [41]. In two recent studies, the pectoralis musculature measured at the T4 level emerged as a prognostic indicator in COVID-19 patients [19,23]. The studies included in this meta-analysis measured pectoralis muscle parameters at the T4 level instead of at the L3 level and examined different pectoralis muscle parameters on chest CT. Chest CT is an important examination for COVID-19 patients on admission, and the PMA, PMI, and PMD can be derived from chest CT parameters. The PMA has emerged as a robust predictor of sarcopenia [42]. Furthermore, research has indicated that decreased PMA and PMI are correlated with adverse prognoses in chronic obstructive pulmonary disease [43], lung cancer [44] and unexplained lung fibrosis [45]. PMD has been employed to investigate the impact of muscular atrophy on chronic obstructive pulmonary disease in mechanically ventilated critically ill patients [43,46]. The present study revealed that COVID-19 patients with a favorable prognosis exhibit high levels of PMD.

The clinical manifestations of COVID-19 range from asymptomatic to mild to severe. Myalgia and generalized weakness have been documented in 25% to 50% of symptomatic COVID-19 patients [47]. Among 214 hospitalized COVID-19 patients in Wuhan, China, 19% exhibited creatine kinase (CK) levels exceeding 200 U/L (a clinically established threshold for elevation), with a maximum value reaching 12,216 U/L [48]. Patients with abnormal imaging findings demonstrate more pronounced clinical manifestations and laboratory abnormalities [49]. There are also case reports regarding rhabdomyolysis associated with COVID-19 [50]. The mechanisms underlying the effects of COVID-19 on skeletal muscle are not fully understood, and several mechanisms have been proposed. COVID-19 is associated with substantial systemic inflammation, with a subset of patients exhibiting a severe cytokine response. Elevated serum levels of inflammatory cytokines, such as TNF-α, have been observed in critically ill COVID-19 patients [51]. TNF-α adversely impacts muscle protein synthesis by reducing mRNA translational efficiency via alterations in eIF-4E availability [52]. In addition, many hospitalized COVID-19 patients experience extended periods of bedrest and limited physical activity. Research indicates that bed rest is associated with decreases in muscle mass, strength, and aerobic exercise capacity [53]. These underlying mechanisms suggest that exploring the potential associations between body composition and COVID-19 outcomes is reasonable.

We acknowledge that this meta-analysis has several limitations. First, the current evidence is limited by the small number of included studies. Second, there was significant heterogeneity between the included studies, which could be due to the quality of the limited number of studies, but further subgroup analysis did not identify the source of heterogeneity. Third, the results are driven by one study with a large sample size.

## Conclusion

In summary, a low PMD is associated with a marginally elevated risk of mortality, whereas a decreased PMI represents a risk factor for intubation in COVID-19 patients. These findings suggest that pectoralis muscle parameters on chest CT may be a useful prognostic tool for COVID-19 patients.

## Supporting information

S1 Table PRISMA 2020 checklist. (DOCX)

S2 Table List of raw analysis data. (XLSX)

S3 Table Review protocol. (XLSX)

S4 Table Newcastle-Ottawa quality assessment scale. (XLSX)
